# The Relevance of Assessing Sagittal Cervical Spine Parameters

**DOI:** 10.7759/cureus.92746

**Published:** 2025-09-19

**Authors:** Sudhir Singh, Neel Mehta, Vibhor Daksh, Vijay P Singh

**Affiliations:** 1 Orthopaedics, Teerthanker Mahaveer Medical College and Research Centre, Moradabad, IND; 2 Radiology, Teerthanker Mahaveer Medical College and Research Centre, Moradabad, IND

**Keywords:** cervical sagittal vertical axis, cervical spine alignment, forward head posture, neck pain, occipito-cervical angle, radiographic analysis

## Abstract

Introduction: Neck pain is a prevalent musculoskeletal condition with a multifactorial etiology, often influenced by biomechanical, occupational, and postural factors. The present study aimed to assess the relationship of cervical alignment parameters, occipito-cervical angle (OC), and C1-C7 cervical sagittal vertical axis (cSVA) with neck pain and demographic variables.

Methods: A prospective observational study was conducted on 255 adult patients of either gender who presented with neck pain at a tertiary care hospital. Radiographic measurements of the Cobb’s angle, OC angle, and cSVA were recorded. Clinical data, including pain type (non-radiating, radiating, radiculopathy/myelopathy), and anthropometric parameters, were also recorded. Statistical analysis was performed using SPSS version 25.0 (IBM Corp., Armonk, NY, USA), with significance set at *p* < 0.05.

Results: The study included 255 subjects (M-102; F-152) with a mean age of 43.91 years and a mean BMI of 24.75 kg/m². The mean Cobb’s angle, OC angle, and cSVA were recorded as 27.24±9.17, 26.78°±3.84, and 28.48±5.42 mm, respectively. No associations were found between these alignment parameters and neck pain, and also with age, gender, and BMI (*p*>0.05).

Conclusion: The findings suggest that occipital and cervical alignment parameters have no relation to neck pain or demographic variables. Assessing these parameters on radiographs may not be useful in routine clinical practice.

## Introduction

The human vertebral column in the sagittal plane has a lordotic curve in the lumbar and cervical regions and a kyphotic curve in the thoracic region. These curvatures distribute the load equally on the vertebral column and are essential for a proper and functional posture [1,2]. Cervical lordosis, thoracic kyphosis, lumbar lordosis, and pelvic incidence are considered interrelated. Any change in one segment may cause reciprocal changes in the adjacent segment to bring the head over the pelvis [3,4]. The cervical spine region has the highest mobility and supports the weight of the head; hence, it is susceptible to many conditions that can significantly affect the quality of life and cause functional disability [3,5]. The disability is often related to compensatory spinal and pelvic posture changes, leading to higher fatigue rates and thereby causing pain [6,7]. Alterations in the cervical curve, such as straightening or kyphosis, have been linked to neck pain, myelopathy, and disability [3]. Recently, neck pain (NP) has become a common musculoskeletal problem, and it ranks fourth in years lived with disability (YLDs) in the global burden of disease [8]. We decided to investigate the connection between neck pain and sagittal head and neck alignment. The occipito-cervical angle (OC), cervical sagittal vertical angle (cSVA), and Cobb’s angle were selected as the sagittal radiographic parameters. We hypothesized that the OC angle, cSVA, and Cobb's angle would correlate with neck pain. The primary objective was to determine the correlation of the OC angle, cSVA, and Cobb’s angle with neck pain. The secondary objective was to determine the correlation of these sagittal parameters with demographic variables such as age, gender, and BMI. 

## Materials and methods

This prospective and cross-sectional (level IV evidence) study was conducted in the orthopedic department of a tertiary health care center. Sample size was determined by Cochran’s statistical formula (n = Z2pq/e2), which suggested 233 cases. Hence, we included 255 cases to overcome the error margin (CI 95%, error 5%). The study was conducted after approval from the College Research Committee (CRC) and Institutional Ethics Committee (IEC) (approval no.: TMU/IEC No. 23/119). Written informed consent was obtained from all subjects. The study was carried out as per the standards laid down in the Helsinki Declaration (1964) and its amendment (2013).

Case selection

The study included 255 adult patients of either gender above 18 years of age with neck pain attending the outpatient department from December 2023 to May 2025. The patients were stratified into three groups. Group I included 110 cases with neck pain without radiation, group II included 113 cases with neck pain with radiation to the shoulder and upper back, and group III included 32 cases with neck pain with radiculopathy/myelopathy. Patients with a history of trauma, previous surgery, inflammatory or infective pathology, degenerative joint disease, and congenital conditions of the cervical spine or shoulder were excluded. Patients were evaluated by detailed history taking, examination, and relevant investigations.

Technique of radiography

A standard lateral view radiograph of the cervical spine was taken with the subject in a comfortable standing position with upper extremities positioned naturally at the side of the body, maintaining horizontal gaze. The OC angle, cSVA, and Cobb’s angle were measured on a radiograph using DICOM software (RediAnt manufactured by Medixant, Poznan, Poland). In cases where radiculopathy or myelopathy was suspected on clinical examination, an MRI of the cervical spine was performed to reach the diagnosis.

Assessment

**Figure 1:**
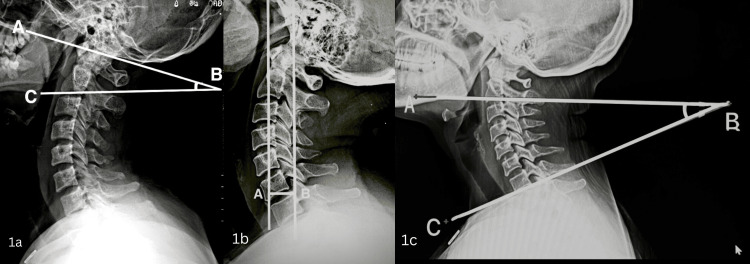
(a) Occipito-cervical angle (OC) angle is the angle between McGregor line (AB) and the tangent on inferior surface of C2 vertebra. (b) Cervical sagittal vertical axis (cSVA) is the horizontal distance between plumb line from tubercle of C1 and the posterior superior corner of C7 vertebra. (c) Cobb’s angle in the angle between the tangent on inferior surface of C2 and the tangent on the inferior surface of C7 vertebra.

The primary outcome measures evaluated in this study were OC angle, cSVA, and Cobb’s angle. The OC angle was measured as an angle between the McGregor line and the tangent line on the inferior surface of the C2 vertebra (Figure 1A) [9]. The cSVA was measured as the horizontal distance between the plumb line dropped from the anterior tubercle of the C1 and the posterior superior corner of the C7 vertebrae (Figure 1B) [9]. The Cobb’s angle was measured as the angle between the tangential line on the inferior surface of the second vertebra (C2) and the tangential line on the inferior surface of the seventh vertebra (C7) (Figure 1C) [10]. All measurements were obtained from lateral radiographs with 100% magnification and analyzed using DICOM software for precision and reproducibility by a radiologist who was blinded to clinical findings. 

Data analysis

Statistical analysis was performed using SPSS software version 25.0 (IBM Corp., Armonk, NY, USA). For comparisons between two groups, an Independent t-test was used, and for multiple group comparisons, Analysis of Variance (ANOVA) was applied. A p-value of < 0.05 was considered statistically significant. All data entry was done in Microsoft Excel (Microsoft, Redmond, WA, USA), and results were compiled for final statistical interpretation.

## Results

The study included 255 subjects (M-102; F-152) with a mean age of 43.91 years and a mean BMI of 24.75 kg/m². Table 1 shows the distribution of cases by age and BMI subcategory. One hundred ten (110) cases reported neck pain with no radiation, radiating pain was reported by 113 cases, and neck pain with radiculopathy was reported by 32 cases.

**Table 1:** Relation of occipito-cervical angle (OC) angle with variables † ANOVA, * Independent t test

	OC angle (^0^)			
	Sub-category/ n	Mean + SD	ANOVA/ t test	P value
Age group	18 to 39 years (99)	27.23 ± 4.28	F=1.405	0.247^†^
	40 to 59 years (101)	26.32 ± 3.56		
	60 to 80 years (55)	26.8 ± 3.47		
	Total	26.78 ± 3.84		
Gender	Female (153)	26.9 ± 3.99	0.605	0.546^*^
	Male (102)	26.6 ± 3.62		
	Total	26.78 ± 3.84		
BMI	Underweight (40)	26.42 ± 3.6	F=1.805	0.167^†^
	Normal (132)	26.75 ± 3.86		
	Overweight (67)	26.35 ± 3.66		
	Obese (16)	26.78 ± 3.84		
	Total	26.78 ± 3.84		
Pain type	Non-radiating (110)	26.47 ± 4.35	F=1.105	0.333^†^
	Radiating (113)	27.18 ± 3.37		
	Myelopathy (32)	26.43 ± 3.52		
	Total	26.78 ± 3.84		

Occipito-cervical angle

The mean OC angle was 26.78±3.84. The OC was recorded as 28.62±6.04, 28.4±4.93, and 28.37±5.18 degrees in the 18-39 year old, 40-59 year old, and 60-80-year old age groups, respectively, with no statistical difference in values among age groups (P=0.247). The values of the angle recorded in males (26.6±3.62) were similar to those in females (26.9±3.99) (P=0.546). In BMI sub-categories, the angle recorded was similar in underweight (27.75±4.1), normal (26.75±3.86), overweight (26.35±3.66), and obese (26.78±3.84) sub-categories (P=0.167). Again, the values in the non-radiating (26.47±4.35), radiating (27.18±3.37), or myelopathic pain group (26.43±3.52) did not differ statistically (P=0.333). The angle remained relatively consistent across all subgroups of age, gender, BMI, and pain types, with a mean of 26.78±3.84° (Table 1).

Cervical sagittal vertical axis

The mean cSVA was 28.48±5.42 mm. The cSVA was recorded as 28.62±6.04, 28.4±4.93, and 28.37±5.18 mm in the 18-39 year old, 40-59 year, and 60-80 year age groups, respectively, with no statistical difference in values among age groups (P=0.247). The values of cSVA recorded in males (29.21±5.8) were similar to females (27.99±5.11) (P=0.08). In BMI sub-categories, the cSVA were similar in underweight (28.22±5.51), normal (28.67±5.7), overweight (28.67±5.7), and obese (28.3±4.95) sub-categories (P=0.84). Again, the values of cSVA in the non-radiating (29.06±5.79), radiating (28.09±5.37), or myelopathic pain group (27.87±4.02) did not differ statistically (P=0.325). The angle remained relatively consistent across all subgroups of age, gender, BMI, and pain types, with a mean of 28.48±5.42 mm (Table 2).

**Table 2:** Relation of cervical sagittal vertical axis (cSVA) with variables † ANOVA, * Independent t test

	cSVA (mm)			
	Sub-category/ n	Mean + SD	ANOVA/ t test	P value
Age group	18 to 39 years (99)	28.62 ± 6.04	F=0.056	0.946^†^
	40 to 59 years (101)	28.4 ± 4.93		
	60 to 80 years (55)	28.37 ± 5.18		
	Total- 255	28.48 ± 5.42		
Gender	Female (153)	27.99 ± 5.11	1.755	0.08^*^
	Male (102)	29.21 ± 5.8		
	Total- 255	28.48 ± 5.42		
BMI	Underweight (40)	28.22 ± 5.51	F=0.174	0.84^†^
	Normal (132)	28.67 ± 5.7		
	Overweight (67)	28.67 ± 5.7		
	Obese (16)	28.3 ± 4.95		
	Total- 255	28.48 ± 5.42		
Pain type	Non-radiating (110)	29.06 ± 5.79	F=1.129	0.325^†^
	Radiating (113)	28.09 ± 5.37		
	Myelopathy (32)	27.87 ± 4.02		
	Total- 255	28.48 ± 5.42		

Cobb’s angle

The mean Cobb’s angle was 27.24±9.17. The Cobb’s angle was 27.56±9.09, 26.5±9.12, and 27.78±9.48 degrees in the 18-39 year, 40-59, and 60-80 year age groups, respectively (p=0.631). The values of Cobb’s angle in males (26.47±9.25) were similar to those of females (28.11±9.05) (P=0.156). In BMI sub-categories, the angle was similar in underweight (26.4±7.85), normal (26.53±9.02), overweight (28.1±10.03), and obese (28±7.43) sub-categories (P=0.61). The values in the non-radiating (27.38±6.87), radiating (27.06±8.53), or myelopathic pain group (27.3±12.58) did not differ among themselves (P=0.325). The angle remained relatively consistent across all subgroups of age, gender, BMI, and pain types (Table 3).

**Table 3:** Relation of Cobb’s angle with variables † ANOVA, * Independent t test

	Cobb’s angle (^0^)			
	Sub-category/ n	Mean + SD	ANOVA/ t test	P value
Age group	18 to 39 years (99)	27.56 ± 9.09	F=0.461	0.631^†^
	40 to 59 years (101)	26.5 ± 9.12		
	60 to 80 years (55)	27.78 ± 9.48		
	Total- 255	27.24 ± 9.17		
Gender	Female (153)	28.11 ± 9.05	1.422	0.156^*^
	Male (102)	26.47 ± 9.25		
	Total- 255	27.24 ± 9.17		
BMI	Underweight (40)	26.4 ± 7.85	F=0.609	0.61^†^
	Normal (132)	26.53 ± 9.02		
	Overweight (67)	28.1 ± 10.03		
	Obese (16)	28 ± 7.43		
	Total- 255	27.24 ± 9.17	F=0.031	0.969^†^
Pain type	Non-radiating (110)	27.38 ± 6.87		
	Radiating (113)	27.06 ± 8.53		
	Myelopathy (32)	27.3 ± 12.58		
	Total- 255	27.24 ± 9.17		

The data was reanalyzed in three different pain subgroups. We did not find any association between these sagittal parameters (Cobb’s angle, OC angle, and cSVA) and pain across age, gender, or BMI subgroups, except in two instances. OC angle differed significantly across age groups in cases of radiating pain (P = 0.026). Similarly, the OC angle shows a significant difference in BMI subgroups in the myelopathy group (P = 0.026). All other comparisons showed no significant differences (Table 4).

**Table 4:** Relation of type of pain with age groups, gender and BMI † ANOVA, * Independent t test

		Non-radiating Pain			Radiating Pain			Myelopathy		
		OC angle (^0^)	cSVA (mm)	Cobb’s angle	OC angle(^0^)	cSVA (mm)	Cobb’s angle	OC angle(^0^)	cSVA (mm)	Cobb’s angle
		Mean + SD	Mean + SD	Mean + SD	Mean + SD	Mean + SD	Mean + SD	Mean + SD	Mean + SD	Mean + SD
Age (years)	18 - 39	26.63 ± 5.08	28.47 ± 6.38	26.73 ± 7.57	28.27 ± 3.48	28.93 ± 6.34	26.89 ± 8.44	25.85 ± 2.67	28.1 ± 3.36	30.88 ± 12.79
	40 - 59	26.35 ± 3.73	29.97 ± 5.38	28.11 ± 6.01	26.41 ± 3.19	27.2 ± 4.17	25.82 ± 8.14	25.92 ± 4.4	26.91 ± 4.31	25.72 ± 13.19
	60 - 80	26.37 ± 4.08	28.34 ± 5.27	28.17 ± 6.08	26.74 ± 3.14	28.22 ± 5.43	29.57 ± 9.18	28.05 ± 3.02	28.96 ± 4.65	25.63 ± 11.55
	F value	0.053	0.941	0.479	3.775	1.131	1.394	1.15	0.643	1.181
	p value	0.948^†^	0.393^†^	0.621^†^	0.026^†^	0.326^†^	0.253^†^	0.331^†^	0.533^†^	0.314^†^
Gender	Female	26.37 ± 4.27	28.71 ± 5.56	26.84 ± 7.15	27.49 ± 3.77	27.58 ± 4.87	27.08 ± 8.55	26.33 ± 3.78	27.29 ± 4.44	24.81 ± 12.94
	Male	26.59 ± 4.49	29.49 ± 6.09	28.05 ± 6.53	26.62 ± 2.42	29.02 ± 6.14	27.03 ± 8.6	26.57 ± 3.24	28.71 ± 3.31	29.65 ± 11.96
	t test	0.258	0.699	0.84	1.478	1.367	0.031	0.194	0.979	;1.555
	p value	0.797^*^	0.486^*^	0.403^*^	0.142^*^	0.174^*^	0.975^*^	0.848^*^	0.336^*^	0.125^*^
BMI	Underweight	28.14 ± 5.07	29.17 ± 6.12	27.67 ± 6.33	26.44 ± 2.63	27.07 ± 5.46	22.22 ± 7.99	29.82 ± 2.86	28.1 ± 3.33	38.4 ± 0
	Normal	26 ± 3.99	28.9 ± 5.86	27.12 ± 7.08	27.77 ± 3.73	28.97 ± 5.93	26.27 ± 8.4	25.61 ± 3.11	26.99 ± 4.18	26.04 ± 12.27
	Overweight	26.31±4.38	29.24±5.68	27.12 ± 7.08	26.54±2.89	27.09±4.16	28.23 ± 9.1	25.72 ± 3.66	29.67 ± 3.94	28.39 ± 13.33
	Obese	26.31 ± 4.38	29.24 ± 5.68	27.62 ± 7.06	26.54 ± 2.89	27.09 ± 4.16	28.94 ± 6.82	25.72 ± 3.66	29.67 ± 3.94	26.78 ± 12.3
	F value	1.77	0.041	0.042	2.016	1.761	1.064	4.126	1.266	0.424
	p value	0.175^†^	0.959^†^	0.989^†^	0.138^†^	0.177^†^	0.368^†^	0.026^†^	0.297^†^	0.736^†^

## Discussion

This research aimed to study the relation of neck pain with sagittal alignment of the cervical spine concerning lordosis (Cobb’s angle) and head posture (OC angle and cSVA). The relation of sagittal parameters with demographic variables such as age, gender, and BMI was also studied. Forward head posture (FHP) is defined as an increased sagittal vertical axis (SVA) in which the head is shifted anteriorly to the shoulder plane compared to the neutral posture [11].

Demographic profile

The variations in lordotic angle in asymptomatic or healthy individuals with age, gender, and BMI have been reported by many published reports. Cervical lordosis increases with age and is more common in males, as has been reported [3,12-15], but there are contradicting reports as well [6,11,16-18]. The data in our study shows no significant difference of occipito-cervical alignments (OC angle and cSVA) as well as in cervical lordosis (Cobb’s angle) with any of the demographic parameters (age, gender, and BMI) (p>0.05). The OC angle in the radiating pain group, among age subgroups, and in the myelopathic pain group, among BMI subgroups, shows significantly different values statistically, but not clinically. The difference of 1-2 mm/degrees in distance and in angles can be disregarded, as this can be just an error of measurement. There is no correlation of BMI in any subcategory of age, gender, and pain as reported earlier as well [16]. It has been reported that obesity influences global spinal parameters rather than cervical measures, with pelvic and lower limb adaptations compensating for added weight [19]. This suggests that the cervical spine remains unaffected by body weight in isolation. Hence, we can safely state that there is no relation of cervical lordosis with age, gender, and BMI.

Sagittal parameters

Lordosis

The relationship between cervical pain and sagittal cervical alignment is not conclusively established [20]. Though it is generally accepted that the cervical spine has a lordotic curve, 35% of the asymptomatic population show a kyphotic curve, and the mere presence of structural variations cannot be termed as an abnormal radiological finding and be linked as a causative factor for neck pain [21]. Further, it has been reported and generally accepted that cervical lordosis depends on thoracic and lumbar lordosis, and changes in the cervical spine occur as a compensatory response to changes in thoracic and lumbar curvature [6]. The “clinically normal” range of cervical lordosis is suggested as 310-400, and lordosis of less than 200 was said to be significantly associated with neck pain [22]. In our study, no case showed lordosis beyond this “clinically normal” range.

Head Posture

Anterior translation of the head in the sagittal plane is called forward head posture and can be measured on a lateral radiograph of the cervical spine by measuring cSVA. There are two methods of determining cSVA. Forward translation is measured by the horizontal distance between the plumb line dropped from the center of the C2 vertebral body (dens) and the posterior superior corner of the C7 vertebra (C2-C7 cSVA). In the second method, the horizontal distance between the plumb line from the anterior tubercle of the C1 vertebra and the posterior superior corner of the C7 vertebra (C1-C7 cSVA) is measured [3]. We have used C1-C7 cSVA values in our study, as they strongly correlate with C2-C7 cSVA (p=0.01) but are not much used [11].

Though correlation of FHP with neck pain has been reported by many authors [9,11,23-25], others have reported that it is contrary to this [26,27]. We, in our study, did not find any relationship of neck pain with or without radiation or with myelopathic changes with cSVA and OC angle (FHP). There was no case of FHP in cases of neck pain reporting to our outpatient department. Recently, it has been reported that FHP is the compensatory change in the cervical spine, secondary to cervical disc degeneration, rather than FHP being the causative factor of disc degeneration [11]. 

Pain Profile

The spine functions as a single unit, and the alignment of the cervical section is influenced by the adjacent spine segment and the overall sagittal balance of the pelvis and spine [3]. The cervical spine supports the weight of the head as well as plays a prominent role in adjusting to thoraco-lumbar malalignment so as to maintain a horizontal gaze [3]. It has been reported that many risk factors may contribute singly or in combination to cause neck pain and disability and, hence, are labeled as a multifactorial disease [28]. The risk factors have been categorized as a) psychological causes (depression, stress, anxiety, cognitive variables, sleep problems, social support, personality, and behavior), b) biological causes (neuromusculoskeletal disorder, autoimmune diseases, genetics, gender, and age), and c) individual causes (work-related, workplace-related, and working posture) [28]. Recently, overuse of computers and mobile phones with improper posture has also been recognized as an individual risk factor. In our study, we did not find any relation of cSVA, OC angle, and Cobb’s angle with pain subtype (radiating, non-radiating, and myelopathic type).

It has been reported earlier as well that the mere presence of structural variations cannot be termed as an abnormal radiological finding and be linked as a causative factor for neck pain [21]. We also believe that correlating clinical symptoms with cervical spine alignment seems appealing but is not easily translatable into clinical practice. Our view is supported by another author who has stated that most risk factors for neck pain are psychosocial and not physical [29,30]. No matter how complex and alluring a computation may be, it is not appropriate to simplify the etiology of neck pain to any form of measurement. Wherever possible, X‑ray studies of the whole spine are done to gain increased understanding of the interplay between the spinal regions rather than looking at a single region on its own.

Limitations

This study had certain limitations. The study relied only on cervical spine sagittal parameters rather than the whole spine in a standing position. Moreover, the study did not control for other potential confounding variables that impact the connection between neck discomfort and head posture.

The findings of the present study suggest that cervical alignment parameters, specifically the Cobb’s angle, OC angle, and cSVA, exhibit limited diagnostic relevance in individuals with neck pain. These results reinforce the concept that neck pain is a multifactorial condition influenced by an interplay of biomechanical, neurological, and psychosocial factors. 

## Conclusions

The OC angle, cSVA, and Cobb’s angle are widely recognized as significant radiological indicators. The data from our study show that these parameters have no relationship with clinical pain types (radiating, non-radiating, and radiculopathy/myelopathy) and demographic factors (age, gender, and BMI) in patients with neck pain. These findings suggest that sagittal measurements of cervical spine radiographs alone have a limited value in predicting the clinical characteristics of neck pain. Hence, the practice of assessing these parameters in routine clinical practice may not be as useful and relevant as generally believed. This implies that further, larger longitudinal studies incorporating psychosocial risk factors and radiography of whole-spine sagittal alignment on their diagnostic and therapeutic utility are required, with a bigger sample size.
